# Supplementary material for: Perception and Performance of Physical Activity Behavior after Head and Neck Cancer Treatment: Exploration and Integration of Qualitative and Quantitative Findings
Source: Int J Environ Res Public Health. 2021 Dec 28;19(1):287. doi: 10.3390/ijerph19010287 (PMC8751059; doi:10.3390/ijerph19010287)
Supplement: Supplementary file 1 [file ijerph-19-00287-s001.zip › Supplementary File S2.pdf]

Supplementary File S2: Overarching Themes of PA Perception Presented with Related Codes, ASE Concepts and Signature Quotes

Theme	Code	Concepts ASE	P28	P29	P30	P31	P32	P33	P34	P35	P36	N	Signature Quote
Barriers and Problems Prioritizing PA	**Physical barriers** Physical limitations may include limitations due to the cancer or it's treatment. In addition they include pre-existing problems.	Barriers/Stimuli	●	●	●	●	●	●	●	●	●	9	P34: *"And making my bed, well in the summer it is easier, because I don't have a heavy duvet blanket on it. But in the winter, I have a woolen duvet, those are very heavy. I really can't do that".*
	**Psychological impact of having cancer**	Barriers/Stimuli	●	●	●	●					●	5	P36*: I just let it (treatment) happen, but that is pretty much all I do."*
	**Fear or insecurities as a result of PA harm**	Barriers/Stimuli	●	●						●	●	4	P29*: I'm not going to do things without thinking it over. I would like to know first whether it is safe"*
	**External Barriers for PA related to prioritizing**. Participants mentioned not being able to prioritize additional PA due to external barriers	Barriers/Stimuli	●	●	●	●	●	●	●			7	P34*: "Badminton; that is what I'd like to do most. But then you need a partner, or join a club. And I can't afford to join a club"*
	**Conditional Intention** Waiting for physical function to improve. Prepared to increase PA after physical barriers are reduced.	Intention	●	●							●	3	P28: *"After that (the cancer) is gone, well, you know, talking and eating is a challenge as well. Still... (hesitates) I think I'll manage then (PA)."*
PA is part of day-to-day life	**Low intensity activities are serving as examples of typical PA behavior**	Knowledge/Skills	●	●	●	●	●	●	●	●	●	9	P31: *"Well, just vacuuming, cleaning the house, clipping something in the garden, mowing the lawn. ... [further explanation]...It's not always to do with attitude, you just do things. .. going upstairs, changing the bed linen... always busy".*
	**Poor awareness of limited capacity to perform higher intensity PA activities**	Behaviour	●	●	●	●	●	●	●	●	●	9	

Supplementary File S2. Continued

Theme	Code	Concepts ASE	P28	P29	P30	P31	P32	P33	P34	P35	P36	N	Signature Quote
PA is part of day-to-day life	**PA is a habit/automatism/natural**	Self-efficacy	●	●	●	●	●	●	●			7	P29: *"...when I go to town for groceries, I do take my bike, actually that's something I simply do as part of normal day to day activities"*
	**PA is important/ a duty**	Behavior			●	●		●	●	●	●	6	P34: "*I just think it (PA) is very important."*
	**Work are important source for PA**	Attitude	●					●	●	●	●	5	P33: *"I work in the mornings. I work in the metal industry. That's pretty hard work, in fact; walking all the time and handling heavy things. I am working with my arms, I am working with my hands and I'm working with my head.*
	**PA is different because of not working**	Knowledge/Skills		●		●					●	3	P31: *"PA is now different. [...] The inevitability of being physically active (for work), that has now changed into needing to plan when you are going to do something.*
	**Sports as example of PA behavior**	Behaviour		●			●					2	
No Need to Increase PA (Lack of Intention)	**No interest or aversion towards more PA**	Attitude	●		●	●	●	●	●	●	●	8	P33: *"I will just continue moving the way I do now. And not with something like that ... [sports]. I think this is sufficient. [...] As long as we keep busy, we're good, I think. No need to add anything much. I'm also physically active at work."*
	**Lack of intention**	Intention	●		●	●			●	●	●	6	P35: *"I just don't take the time for it (PA), or don't want to make time .... After I have done my work - I think that's enough obligatory physical activity - then I'd rather read a book ... or watch something on television that interests me."*

Supplementary File S2. Continued

Theme	Code	Concepts ASE	P28	P29	P30	P31	P32	P33	P34	P35	P36	N	Signature Quote
PA is Associated with Positive Feelings or Effects	**Positive feelings effects as stimuli for PA**	Barriers/Stimuli	●	●	●	●	●	●	●	●		8	partner P32: *" Yes, you enjoy doing it (PA). If you like doing something, you keep doing it"*
	**Relating PA to positive feelings/effects**	Attitude		●	●	●	●	●	●	●		7	P33: *"But it is also pleasant to be outside, to take a stroll. My wife spends the whole day in the hospital, but then we say 'let's catch some fresh air"*
	**Committed to (restoring) PA habits**	Intention		●			●	●	●			4	P32: *"I was first operated on in September, in October a second time, and in November a third time and then my husband wouldn't let me go work out any sooner than January. So I started on January 2nd, right away."*
	**Understanding the health benefits of PA**	Knowledge/Skills		●				●	●		●	4	P34: *"For example, this morning I washed the dishes and mopped the kitchen; and just by getting moving, the pain [in my knees] is less bad".*
	**Intrinsically motivated for PA**	Self-efficacy				●	●		●			3	
Limited Social Support and Persuasion	**PA advise by physicians/nurses little, none or no recalling by participant**	Social norm	●		●	●		●	●	●	●	7	P33: *"Well wait a minute - I believe they did tell me once to 'keep moving'. But I think I mentioned it first. That I'm usually active. Yes, and then they told me that I should keep moving."*
	**Social network stimulates PA and/or shows some fear of harm**	Social norm	●	●		●	●	●	●			6	P29: *"But when I'm with colleagues - these people are very much into exercising, and someone will ask you at some point. "What time did you run?"...that can be very stimulating".* P32: *"I would have liked to start [working out] a little sooner, but he [points at partner] wouldn't let me that is why. But otherwise nothing has changed".*
	**Physiotherapist is needed or involved**	Social norm		●	●			●		●	●	5	
	**Need to do it yourself/to have control**	Attitude	●	●		●			●	●		5	P35: "*I would prefer to build myself a (PA) schedule".*
	**No need for professional help with PA**	Self-efficacy	●			●			●	●		4	P31: *"But, look, I have always done that [PA] myself. I was a single mom, so I took all my decisions myself. I know very well what I can do and what is good for me. I really need no help with that.*
