# Supplementary material for: Perception and Performance of Physical Activity Behavior after Head and Neck Cancer Treatment: Exploration and Integration of Qualitative and Quantitative Findings
Source: Int J Environ Res Public Health. 2021 Dec 28;19(1):287. doi: 10.3390/ijerph19010287 (PMC8751059; doi:10.3390/ijerph19010287)
Supplement: Supplementary file 1 [file ijerph-19-00287-s001.zip › Supplementary File S1.pdf]

Supplementary File S1: Sample questions from the topic list.

Domain<br>ASE model	Sample question
**Knowledge**	How would you define physical activity?<br>What does a day look like for you?<br>What kind of activities are you usually doing in and around the house?<br>How much exercise or sport do you have now?
**Attitude**	How do you feel about physical activity? Why?<br>What are your preferences regarding physical activity?<br>Do you prefer physical activity individually or in a group?<br>Do you prefer physical activity at home or in a sports facility?<br>Do you prefer physical aca (sports) activity or by engaging in daily activities/hobbies?<br>Do you think you should be physically active / you should have exercise?
**Barriers**	What are the reasons for you to be less physically active?<br>Do you have symptoms that you think affect physical activity?<br>If so, what kind of symptoms?<br>If so, to what extent?<br>If so, how does this affect the extent to which you move?<br>On what kind of days is it more difficult for you to move?
**Stimuli**	What are reasons for you to be more physically active?<br>On what kind of days is it easier for you to be physically active?
**Self-efficacy**	Do you see opportunities to be physically active or exercise in your environment?<br>What kind of opportunities do you see?<br>What opportunities are you missing?<br>What does a day look like for you?<br>What kind of activities are you usually doing in and around the house?<br>Are you consciously thinking about physical activity?<br>If so, what are you thinking about?<br>If so, does this affect the degree of activity?<br>If so, does this affect the type of activity?<br>What are your strategies for solving barriers?<br>Do you think you can overcome the barriers? If so, how?<br>Who or what would help you to be physically active on difficult days? Why?<br>How do you ensure that you still are active on days when it is more difficult for you (barriers)?<br>Are you unsure about anything related to physical activity?
**Intention**	What makes you physically active?<br>How often would you like to be active (ideal physical activity pattern)?<br>Do you achieve this ideal physical activity pattern?

Domain<br><br>ASE model	Sample question
	What makes you achieve this or not? (self-efficacy)<br>Is being physically active important to you? Why?<br>What are your personal preferences regarding physical activity?<br>If barriers for physical activity are mentioned:<br>What would you do if the barriers were not present?<br>Do you have any influence on this? (self-efficacy)
**Social influence and social support**	Do you receive support or guidance in physical activity?<br>If so, by whom?<br>If so, does this affect the degree of activity?<br>If so, does this affect the type of activity?<br>How do you think about support or guidance in physical activity?<br>During the process of diagnosis, treatment and aftercare by health care professionals, has physical activity been discussed with you?<br>If so, by what kind of health care professionals?<br>If so, what was discussed with you?<br>Does this affect or did this affect the degree of physical activity?<br>Does this affect or did it affect the type of physical activity?<br>Looking back, would you have wanted to receive more support/advice and in what phase of the treatment?<br>If so, how could this have affected physical activity?<br>How would you feel about receiving (possibly extra) guidance in physical exercise?<br>What do you think this guidance should consist of?<br>Who do you feel should do this?<br>In what way?<br>How will this affect your degree of physical activity?<br>In what way are the people around you physically active?<br>What type of physical activity are they doing?<br>To what extend are they physically active?<br>Does this affect the extent to which you are physically active? (if so, what kind of influence?)<br>Does this affect the type of your physical activity you have? (if so, how?)<br>How does this affect your choices regarding physical activity?
